# Correction: Use of IoT sensing and occupant surveys for determining the resilience of buildings to forest fire generated PM_2.5_

**DOI:** 10.1371/journal.pone.0225492

**Published:** 2019-11-14

**Authors:** Jovan Pantelic, Megan Dawe, Dusan Licina

There are errors in the Author Contributions. The correct contributions are: Conceptualization: JP MD DL. Data curation: JP MD DL. Formal analysis: JP MD DL. Funding acquisition: JP. Investigation: JP MD DL. Methodology: JP MD DL. Project administration: JP. Resources: JP. Supervision: JP. Writing–original draft: JP MD DL. Writing–review & editing: JP MD DL.
