# Supplementary figures and images for: Use of IoT sensing and occupant surveys for determining the resilience of buildings to forest fire generated PM2.5
Source: PLoS One. 2019 Oct 16;14(10):e0223136. doi: 10.1371/journal.pone.0223136 (PMC6795448; doi:10.1371/journal.pone.0223136)

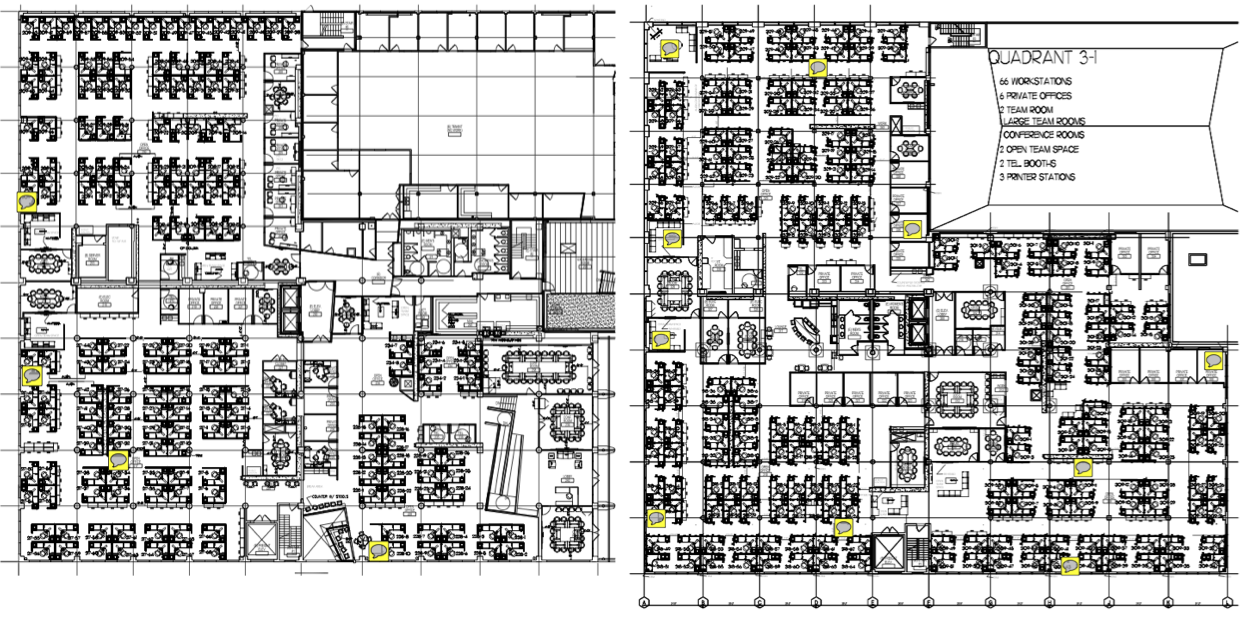

Supplement: S1 Fig — Distribution of sensors in the 4th Street Building a) floor 2; b) floor 3. (TIFF) [file pone.0223136.s001.tiff]

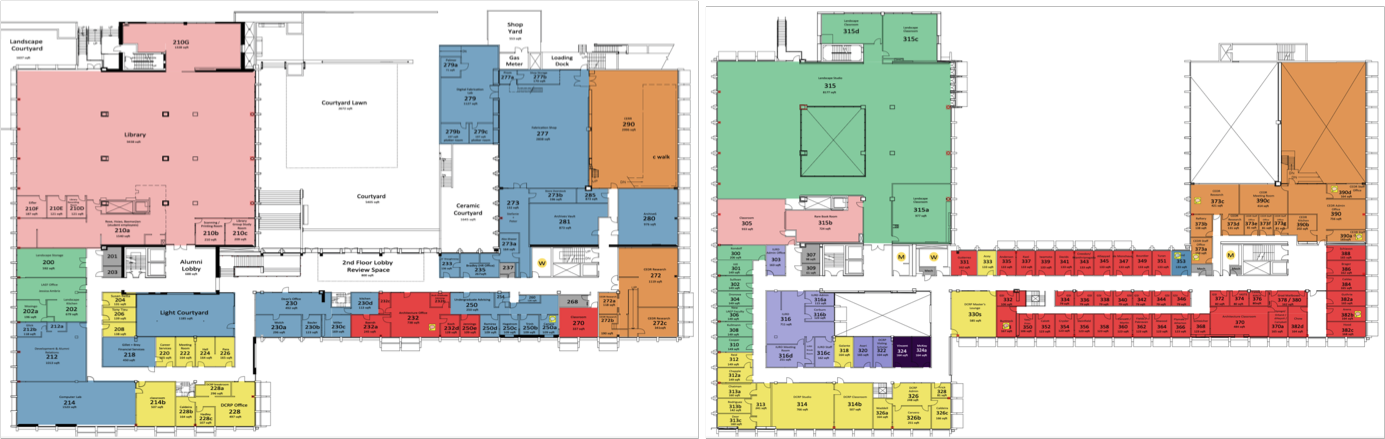

Supplement: S2 Fig — Distribution of sensors in the Wurster Hall a) floor 2; b) floor 3. (TIFF) [file pone.0223136.s002.tiff]

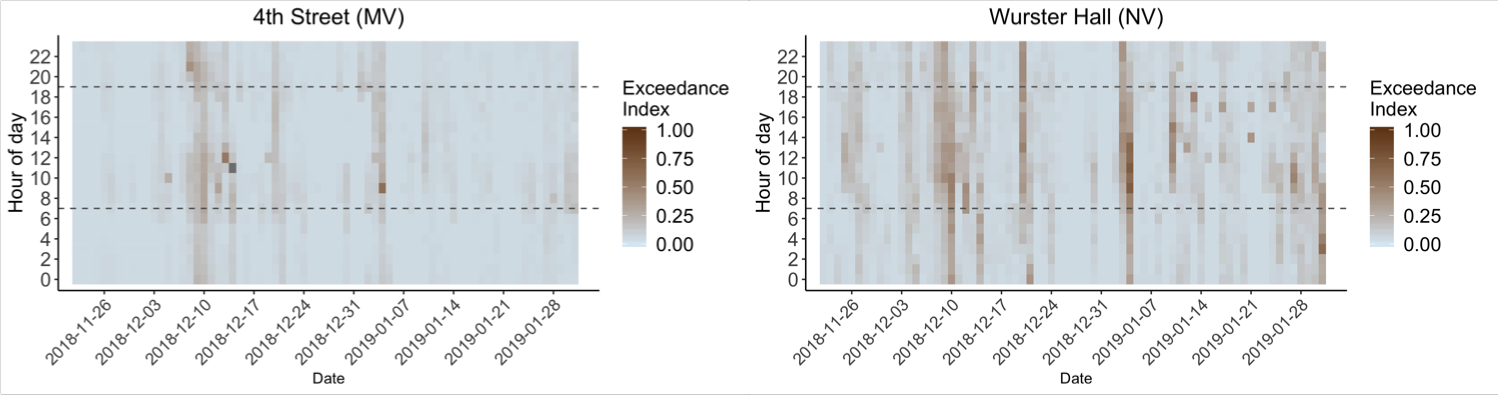

Supplement: S3 Fig — (TIFF) [file pone.0223136.s003.tiff]
