# Supplementary material for: Use of IoT sensing and occupant surveys for determining the resilience of buildings to forest fire generated PM2.5
Source: PLoS One. 2019 Oct 16;14(10):e0223136. doi: 10.1371/journal.pone.0223136 (PMC6795448; doi:10.1371/journal.pone.0223136)
Supplement: Attachment — Submitted filename: Response to Reviewers.docx [file pone.0223136.s005.docx]

**Response to Reviewers**

**Journal Requirements:**

1. We note that Figure 2 in your submission contain [map/satellite] images which may be copyrighted. All PLOS content is published under the Creative Commons Attribution License (CC BY 4.0), which means that the manuscript, images, and Supporting Information files will be freely available online, and any third party is permitted to access, download, copy, distribute, and use these materials in any way, even commercially, with proper attribution. For these reasons, we cannot publish previously copyrighted maps or satellite images created using proprietary data, such as Google software (Google Maps, Street View, and Earth). For more information, see our copyright guidelines: <http://journals.plos.org/plosone/s/licenses-and-copyright>.

Answer: Figure 2 is removed from the manuscript as we didn’t see too much of its use.

2. Please include additional information regarding the survey or questionnaire used in the study and ensure that you have provided sufficient details that others could replicate the analyses. For instance, if you developed a questionnaire as part of this study and it is not under a copyright more restrictive than CC-BY, please include a copy, in both the original language and English, as Supporting Information.

Answer: Thank you for the useful suggestion - the full survey questionnaire is included in the Supporting Information document.

3. Please amend your current ethics statement to confirm that your named institutional review board or ethics committee specifically approved this study.

Answer: In section 2.3.4 sentence is included: The University of California at Berkeley Institutional Review Board approval was obtained Protocol ID:2019-02-11802.

4. Have the authors made all data underlying the findings in their manuscript fully available?

Reviewer #1: No

Answer: All the data from the study is now included.

**General Comments**

**Reviewer #1:** In general the content of the paper is written in a good flow. The Results and conclusions meet the objectives stated in the Introduction. The methodology is strengthened by benchmarking with the standards used in the actual operation.

Answer: Thank you for your comments.

**Reviewer #2:**

1. Weakness of Paper

Yet, while there are some merits of the study as mentioned above, this study needs some improvements. There is a need to highlight the implication of this study. The introduction section needs to be more concise in terms of elaborating on the research gap.

Answer: The introduction section has been revised and is more concise now. The research gap is explained better. All the changes are kept as track changes in the manuscript.

2. Use of Language

The paper is well written. Language is easy to understand and explains the matter correctly. However, some sentences are too long, making the reader hard to understand. There are some typos need to be fixed (for example see 2.4.3 paragraph 2 line 7).

Answer: Long sentences have been identified and rewritten. You can see them as track changes in the manuscript.
